# Harmful and Beneficial Role of ROS

**DOI:** 10.1155/2016/7909186

**Published:** 2016-10-12

**Authors:** Sergio Di Meo, Tanea T. Reed, Paola Venditti, Victor M. Victor

**Affiliations:** ^1^Dipartimento di Biologia, Università di Napoli “Federico II”, 80126 Napoli, Italy; ^2^Department of Chemistry, Eastern Kentucky University, Richmond, KY 40475, USA; ^3^Service of Endocrinology, Dr. Peset University Hospital, Foundation for the Promotion of Health and Biomedical Research in the Valencian Region (FISABIO), 46010 Valencia, Spain

Reactive oxygen species (ROS) are an unavoidable byproduct of oxygen metabolism and their cellular concentrations are determined by the balance between their rates of production and their rates of clearance by various antioxidant compounds and enzymes. For a long time ROS were thought to cause exclusively toxic effects which were associated with various pathologies, including carcinogenesis, neurodegeneration, atherosclerosis, diabetes, and aging. However, to date, it is known that while prolonged exposure to high ROS concentrations may lead to various disorders, low ROS concentrations exert beneficial effects regulating cell signaling cascades.

The papers reported in this issue focus attention on some aspects of ROS biology including the impact of ROS production on various body districts and the defense arising from endogenous and exogenous antioxidants, beneficial effects of ROS production, and ROS regulation of signaling pathways. However, the examination of the manuscripts clearly shows that the passage of time has partly changed the approach to various topics. For example, although in some works different substances, including antioxidants, have yet been used, in other works different procedures and substances, including products or extracts, have been successfully used in the treatment of oxidative stress and related disorders due to the complex bioactive compounds they contain.

Thus, A. V. Maksimenko studied the effects of intravenous injection of the superoxide dismutase-chondroitin sulfate-catalase (SOD-CHS-CAT) conjugate in a rat model of endotoxin shock. In this way he demonstrated the effectiveness of the conjugate in prevention and medication of oxidative stress damage, which is only partly due to prevention of NO conversion in peroxynitrite.

The review of V. D. Prokopieva et al. examined properties and biological effects of the antioxidant carnosine and presented data on successful use of carnosine in different pathologies. Such data show that carnosine is an effective antioxidant able to protect tissues against various adverse factors inducing development of oxidative stress.

S. Ponist et al. evaluated the therapeutic potential of carnosine in rat adjuvant arthritis. The results obtained on two animal models (model of local acute inflammatory reaction and subchronic model of rodent polyarthritis) showed that carnosine had systemic anti-inflammatory activity and protected rat brain and chondrocytes from oxidative stress.

A. Matuszyk et al. found that administration of exogenous obestatin accelerates the healing of acetic acid-induced colitis, an effect partly due to anti-inflammatory properties of obestatin that reduces IL-1*β* concentration and myeloperoxidase activity in colonic mucosa.

C. Liu et al. used the aqueous extract of* Cordyceps militaris* fruit body in streptozotocin-induced diabetic rats and found that the extract displays antidiabetic and antinephrotic activity due to its ability to attenuate oxidative stress.

W. J. Bae et al. examined the effects of decursin extracted from* Angelica gigas* Nakai (AG) on antioxidant activity* in vitro *and in a cryptorchidism-induced infertility rat model. Their study suggests that decursin is able to reduce oxidative stress by Nrf2-mediated upregulation of heme oxygenase-1 (HO-1) in rat experimentally induced unilateral cryptorchidism and may improve cryptorchidism-induced infertility.

E. Kerasioti et al. studied the protective effect of sheep whey protein (SWP) against tert-butyl hydroperoxide- (tBHP-) induced oxidative stress in endothelial cells. Their findings demonstrate that SWP protects endothelial cells from oxidative stress increasing GSH levels and decreasing GSSG, lipid peroxidation, protein oxidation, and ROS levels.

P. Boonruamkaew et al. studied the effect of an antioxidative nanoparticle (RNP^N^) that they recently developed against APAP-induced hepatotoxicity in mice. Their findings lead to concluding that RNP^N^ possesses effective hepatoprotective properties and does not exhibit the notable adverse effects associated with NAC treatment.

M. J. Gomes et al. evaluated the influence of exercise on functional capacity, cardiac remodeling, and skeletal muscle oxidative stress in rats with aortic stenosis- (AS-) induced heart failure (HF). They found that exercise improves functional capacity in rats regardless of echocardiographic parameter changes. In soleus, exercise reduces oxidative stress, preserves antioxidant enzyme activity, and modulates mitogen-activated protein kinases (MAPK) expression

S. Kremserova et al. evaluated the role of myeloperoxidase (MPO) in the regulation of acute lung inflammation and injury. They showed that MPO deficiency enhances neutrophilia during LPS-induced airway inflammation due to altered accumulation of proinflammatory cytokine RANTES (regulated on activation, normal T cell expressed and secreted) and reduces cell death of MPO deficient neutrophils. The role of MPO in the regulation of the course of pulmonary inflammation, independent of its putative microbicidal functions, can be potentially linked to its ability to modulate the life span of neutrophils and affect accumulation of chemotactic factors at the site of inflammation.

J. Petrović et al. investigated whether magnesium supplementation in sedentary and rugby players young men could protect peripheral blood lymphocytes (PBL) from hydrogen peroxide-induced DNA damage. They found that magnesium supplementation has marked effects in protecting the DNA from oxidative damage in both men with different lifestyles.

T. Kataoka et al. compared the mitigating effects on chronic constriction injury- (CCI-) induced neuropathic pain of radon inhalation and pregabalin administration and examined the combination effects of the treatments. They found that combined effect of radon and pregabalin is an additive effect because it has mitigative effect similar to the effects of remarkably higher dose of pregabalin. The possible mechanism is the activation of antioxidative functions induced by radon inhalation.

G. Espinha et al. found that the inhibition of RhoA GTPase, an enzyme overexpressed in highly aggressive metastatic tumors, increases sensitivity of melanoma cells to UV radiation effects, suggesting that this GTPase represents a potential inhibitory target for metastatic melanomas.

Some works have addressed the problem of the dual role played by ROS or ROS producing enzymes.

N. Kaludercic and V. Giorgio described mitochondria as a major site of production and as a target of ROS/RNS and discussed how the posttranslational modifications of ATP synthase due to ROS/RNS generation might play a dual role by promoting cell death or survival depending on their relative effects on mitochondrial ATP synthase catalysis and PTP.

H. Pei et al. summarized the present understanding of the role played by mitochondrial functional proteins such as electron transport chain complexes, uncoupling proteins, mitochondrial dynamic proteins, translocases of outer membrane complex, and mitochondrial permeability transition pore in ROS production and in protection of mitochondrial integrity and function in ischemic heart diseases.

M. G. Battelli et al. reviewed the physiological and pathological roles of xanthine oxidoreductase- (XOR-) derived oxidant molecules showing that they may result in either harmful or beneficial outcomes. Indeed, XOR generates free radicals which are responsible for tissue damage in hypoxia/reoxygenation and ischemia/reperfusion, have proinflammatory activity, are involved in cancer pathogenesis, and favor the progression to malignancy by inducing angiogenesis and cell migration. On the other hand, XOR products may activate the expression of the proapoptotic protein p53 and transcription factors with antitumorigenic and antiproliferative activity.

H.-Y. Tan et al. reviewed the role of ROS in maintaining the homeostatic functions of macrophage and in particular macrophage polarization. They also reviewed the biology of macrophage polarization and the disturbance of the balance of the different functional phenotypes in human diseases.

J. A. Hernández et al. reviewed the role of lipids in the neuronal damage induced by ethanol-related oxidative stress and in the related compensatory or defense mechanisms. They showed that ethanol-induced neurodegeneration is at least partly the result of the equilibrium between the toxicity of signaling lipids and the protection that some lipids, such phosphatidylethanolamine and cholesterol, confer to the cell.

A. Schmidt et al. used a HaCaT keratinocyte cell culture model to investigate redox regulation and inflammation to periodic, low-dose oxidative challenge generated by recurrent incubation with cold physical plasma-treated cell culture medium. They investigated the HaCaT keratinocyte global transcriptomic profile over three months to identify genes responsible for adaptations to periodic oxidative stress as seen in redox-related diseases of the skin. Their results suggest that all keratinocytes may have adapted to redox stress over time, significantly altering their basal gene expression profile.

E. Ershova et al. studied the influence of a water-soluble fullerene derivative (F828) on serum-starving human embryo lung diploid fibroblasts HELFs. They found that F828 exerts a block on genotoxic effect of oxidative stress in serum-starving HELFs. The decrease in the number of double strand breaks and apoptosis was maximum at concentrations 0.2–0.25 *μ*M, whereas, at concentrations higher than 0.5 *μ*M, excessive ROS scavenging was accompanied by increased cell death rate.

The problem of the role of reactive species sources in health and disease was examined by S. Di Meo et al. They, after examining the cellular localization and supposed involvement of such sources in tissue dysfunction and protection, examined experimental evidence concerning their harmful and protective effects in a normal physiological activity, such as exercise, and in pathologic conditions, such as diabetes and neurodegenerative diseases.

Some works have faced different problems that are still united by the oxidative stress impact on various pathological conditions and the factors involved in the signaling pathways

N. T. Costa et al. evaluated the involvement of TNF-*α* and insulin resistance (IR) in the inflammatory process, oxidative stress, and disease activity in patients with rheumatoid arthritis (RA). They demonstrated that IR and TNF-*α* are important factors involved in redox imbalance in patients with RA which seems to be due to the maintenance of inflammatory state and disease activity.

S. Vranková et al. studied the effects of NF-kB inhibition on ROS and NO generation and blood pressure (BP) regulation in hereditary hypertriglyceridemic rats. They found that NF-kB inhibition leads to decreased ROS degradation by SOD followed by increased heart oxidative damage and BP elevation despite the increase in eNOS protein.

L. Minutoli et al. described the current knowledge on the role of NLRP3 inflammasome in some organs (brain, heart, kidney, and testis) after I/R injury, with particular regard to the role played by ROS in its activation. They conclude that a definite comprehension of the role of NLRP3 inflammasome in the host responses to different danger signals is still lacking.

S. Kazemi et al. investigated the effects of the xenoestrogenic chemical Bisphenol A (BPA) on hepatic oxidative stress-related gene expression in rats. Their finding demonstrated that BPA generates ROS and increases the expression of HO-1 and Gadd45b genes that cause hepatotoxicity.

A. N. Onyango reviewed the potential pathways of the endogenous formation of singlet oxygen and ozone, their relevance to human health, and how dietary factors affect generation and activity of such oxidants.

E. Gammella et al. described the multifaceted systems regulating cellular and body iron homeostasis and discussed how altered iron balance may lead to oxidative damage in some pathophysiological settings.

X. Wang et al. found that the transcription factor BTB and CNC homology 1 (Bach1) induces endothelial cell apoptosis and cell cycle arrest through ROS generation and, consequently, that functional downregulation of Bach1 may be a promising target for the treatment of vascular diseases.

M. Nita and A. Grzybowski, examining the current literature, showed that excessive production of reactive oxygen species and oxidative stress play important role in the pathogenesis of many age-related ocular diseases and other pathologies of the anterior and posterior eye segment in adults. ROS stimulate cells' death via apoptosis process, participate in the activation of proinflammatory and proangiogenic pathways, and are associated with the autophagy process.

T. R. S. Hamilton et al. evaluated lasting effects of heat stress-induced oxidative stress on ejaculated and epididymal sperm and found that it leads to rescuable alterations after one spermatic cycle in ejaculated sperm and also after 30 days in epididymal sperm.

M. L. Fanjul-Moles and G. O. López-Riquelme examined the relationships between oxidative stress, circadian rhythms, and retinal damage in humans, particularly those related to light and photodamage. Their review highlights the role of oxidative stress as one of the main causes of age-related macular degeneration (AMD) etiologies, a disease to which, in addition to genetic predispositions, at least four processes contribute: lipofuscinogenesis, drusogenesis, local inflammation and neovascularization, and immunological mechanisms.

H. Nagahisa et al. studied structural and functional changes induced by SOD1 deficiency and their results suggested that muscle is damaged by ROS produced in the TypeIIx/b fibers of the gastrocnemius muscle, accelerating the proliferation and differentiation of satellite cells in SOD1 KO mice.

It is our opinion that the articles included in this special issue, despite dealing with so different topics, represent an important contribution to the knowledge of the harmful and beneficial effects of ROS in living organisms.

*Sergio Di Meo* *Sergio Di Meo* *Tanea T. Reed* *Tanea T. Reed* *Paola Venditti* *Paola Venditti* *Victor M. Victor* *Victor M. Victor*

